# The Complete Chloroplast Genome of Water Crowfoot of *Ranunculus* cf. *penicillatus* and Phylogenetic Insight into the Genus *Ranunculus* (sect. *Batrachium*)

**DOI:** 10.3390/ijms26146953

**Published:** 2025-07-20

**Authors:** Jurgita Butkuvienė, Donatas Naugžemys, Donatas Žvingila

**Affiliations:** 1 Institute of Biosciences, Life Sciences Center, Vilnius University, Sauletekio av. 7, 10257 Vilnius, Lithuania; jurgita.butkuviene@gmc.vu.lt; 2 Botanical Garden, Vilnius University, Kairenu Str. 43, 10239 Vilnius, Lithuania; donatas.naugzemys@gf.vu.lt

**Keywords:** *Ranunculus penicillatus* group, *Batrachium*, aquatic plants, chloroplast, phylogenetic analysis

## Abstract

This study describes the first complete chloroplast genome of *Ranunculus* cf. *penicillatus* and provides new insights into the genetic composition and evolutionary relationships of the *Ranunculus* genus. The genome was assembled and characterized using high-throughput sequencing technologies, revealing a circular structure encompassing 158,313 base pairs. Comparative analysis with the chloroplast genomes of related species within the *Ranunculus* genus highlights notable variations in structural organization, which can elucidate potential adaptive evolutionary mechanisms. Phylogenetic analyses conducted using the maximum likelihood approach resulted in the placement of *Ranunculus* cf. *penicillatus* within a well-defined clade, revealing its relationship with other taxa. This study not only enriches the existing plastid genomic data of the genus *Ranunculus* but also serves as an additional resource for future studies on the phylogenetics, systematics, and conservation biology of this diverse group of aquatic plants. The findings highlight the importance of complete chloroplast genomes in the *Ranunculus* section *Batrachium*, an evolutionarily young group of aquatic plants, for understanding plant diversity and evolution. The genome can be accessed on GenBank with the accession number PV690257.

## 1. Introduction

The genus *Ranunculus* L. (Ranunculaceae), which includes more than 600 species worldwide, represents one of the most taxonomically and ecologically diverse lineages of flowering plants [1,2]. Among its complex taxonomic subdivisions, the section *Batrachium* (DC.) S.F. Gray, commonly known as water crowfoot, is one of the most difficult taxonomic treatment groups of aquatic plants. This group covers aquatic and semiaquatic species that play essential functions in freshwater ecosystems across Europe and parts of Asia. However, understanding phylogenetic relationships within this group and, more broadly, across the genus has long been complicated by phenotypic convergence, hybridization, and limited genomic data.

Chloroplast genomes provide valuable sources of molecular markers for plant phylogenetic analysis because of their relatively conserved structure, uniparental inheritance, and abundant phylogenetic signals [3,4]. In recent years, complete sequencing of chloroplast genomes has emerged as a powerful instrument for clarifying evolutionary relationships within complex plant groups [5]. Despite this advancement, comprehensive chloroplast genomic data for the *Ranunculus* section *Batrachium* continue to be limited, particularly for plants of *Ranunculus* cf. *penicillatus*, which exhibit not only capillary leaves but also floating or intermediate leaf forms [2].

In this study, we present the first fully sequenced chloroplast genome of a representative water crowfoot belonging to the *R.* cf. *penicillatus*. We conducted a comparative analysis with available chloroplast genomes of related species within the *Ranunculus* genus, highlighting notable variations in structural organization, which may be essential in elucidating potential adaptive evolutionary mechanisms. Phylogenetic analyses carried out using the maximum likelihood approach locate *R.* cf. *penicillatus* within a well-defined clade, indicating its relationship with other taxa. This study not only enriches the existing plastid genomic data of the genus *Ranunculus* section *Batrachium* but also serves as an additional resource for future studies on the phylogenetics, systematics, and conservation biology of this diverse group of submerged macrophytes. This study highlights the importance of complete chloroplast genomes in the *Ranunculus* section *Batrachium*, an evolutionarily young group of aquatic plants, for understanding plant diversity and evolution.

## 2. Results

**Figure 1:**
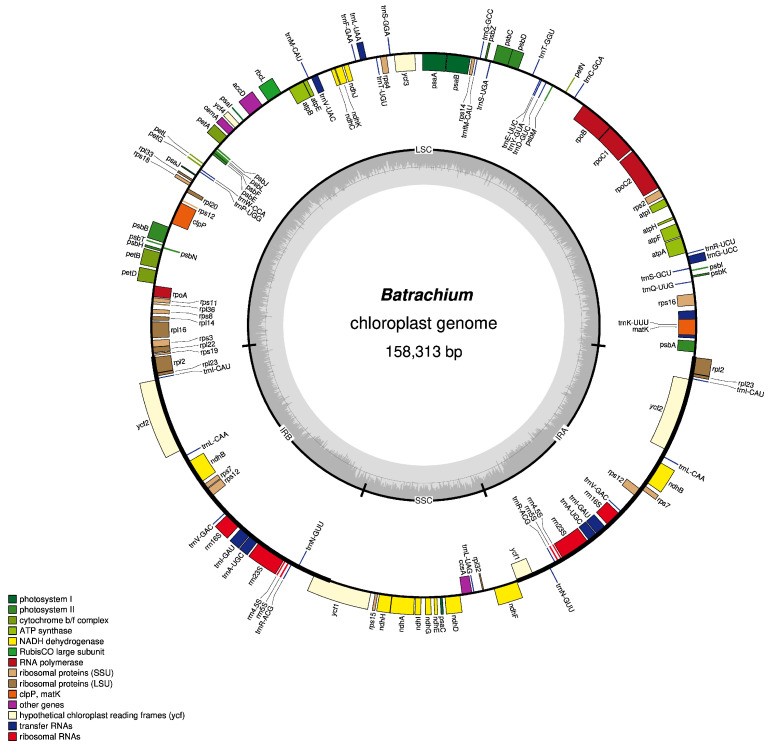
Map of the chloroplast genome of *Ranunculus* section *Batrachium* (*Ranunculus* cf. *penicillatus*). The genes located inside and outside the circle are transcribed in the clockwise and counterclockwise directions, respectively. Genes belonging to different functional groups are represented in different colors. The thick lines indicate the extent of the inverted repeats (IRa and IRb) that separate the genomes into small single-copy (SSC) and large single-copy (LSC) regions. The innermost darker gray corresponds to the GC content, whereas the lighter gray corresponds to the AT content.

The chloroplast genome of a plant from the *Ranunculus* cf. *penicillatus* group is structured as a typical circular, double-stranded molecule with a length of 158,313 bp (GenBank accession no. PV690257) and an overall GC content of 36.80% (Figure 1). The large single-copy (LSC) region is 84,937 bp long, and the short single-copy (SSC) region is 17,635 bp long. Each of the inverted repeat (IR) regions measures 27,849 bp in length. The reported Chloroplast genome contains 112 genes, including unique genes and genes that are duplicated in the IRs. The group of 112 unique genes includes 78 protein-coding genes, 30 tRNA genes, four rRNA genes, and nine conserved chloroplast open reading frames (ORFs).

**Figure 2:**
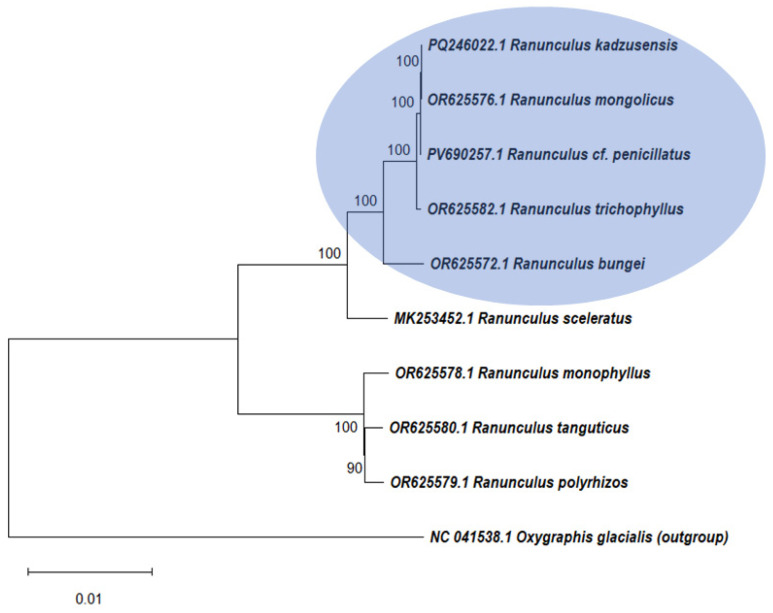
Phylogenetic tree comprising nine species in the family Ranunculaceae using the maximum likelihood (ML) method, which is based on complete chloroplast genome sequences. The numbers above the nodes represent the bootstrap support value for each branch. Aquatic plants are marked in blue.

Phylogenetic reconstruction revealed that the genome of *Ranunculus* cf. *penicillatus* is closest to the genomes of *R. mongolicus*, *R. kadzusensis,* and a plant from the *R. trichophylus* group, according to the GenBank database (Figure 2). All aquatic *Ranunculus* form a separate clade (marked in blue) from terrestrial *Ranunculus* species (Figure 2).

All *Ranunculus* species exhibit a typical quadripartite chloroplast genome structure, comprising a large single-copy (LSC) region, a small single-copy (SSC) region, and two inverted repeats (IRs) (Figure 1).

The lengths of all the compared chloroplast genome sequences of Ranunculaceae ranged from 155,973 bp (*R. monophyllus*) to 158,314 bp (*R. trichophyllus*), with overall GC contents ranging from 36.7 to 41.22% (Table 1). In the chloroplast genome sequences of samples from the genus *Ranunculus*, a total of 112 genes were identified, 78 of which were protein-coding genes. The chloroplast genome of *Ranunculus* cf. *penicillatus* shows identical results, also featuring 112 genes, including 78 that are protein-coding (see Table 1).

**Figure 3:**
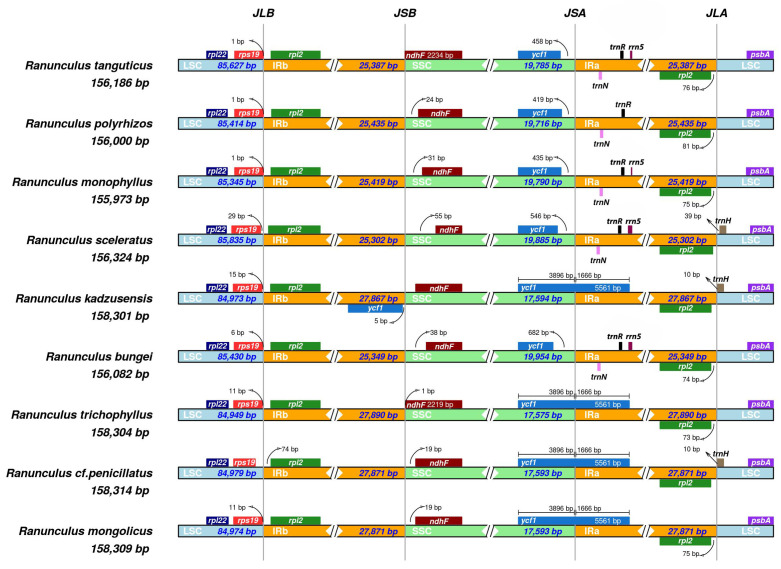
IR scope analysis of the nine *Ranunculus* chloroplast genomes.

In the samples from the genus *Ranunculus*, all the chloroplast genome sequences contained an LSC region measuring between 84,937 and 85,840 base pairs (bp), an SSC region ranging from 17,635 to 19,956 bp, and a pair of IR regions measuring between 25,302 and 27,853 bp. This structure is typical among angiosperms (Table 1). Notably, four species from the genus *Ranunculus*–*R. mongolicus*, *R. trichophyllus*, *R. kadzusensis*, and *R.* cf. *penicillatus*, presented an extended IR region (Figure 3).

In these species, a large portion of the *ycf1* gene (3986 bp + 1666 bp) is duplicated within both IR regions, indicating a significant IR expansion into the SSC region. In contrast, species such as *R. tanguticus*, *R. polyrrhizos*, *R. monophyllus*, and *R. sceleratus* exhibited much shorter *ycf1* or *ndhF* segments at the IR boundaries, suggesting no substantial IR expansion (Figure 3). *R. bungei* also shows evidence of IR expansion, though it to a lesser extent compared to *R. kadzusensis*, *R. trichophyllus*, *R*. cf. *penicillatus*, and *R. mongolicus*.

## 3. Discussion

The Ranunculaceae family exhibits remarkable diversity, encompassing approximately 650 species with a global distribution [7]. Among them, the genus *Ranunculus* is one of the most taxonomically complex groups and is often characterized by convergent evolution, polyploidy, and frequent hybridization, particularly within aquatic lineages such as the section *Batrachium* [8,9,10,11,12]. These complexities have challenged taxonomists for decades and have hindered the establishment of a stable classification system for many species in the group [13,14,15,16,17,18,19].

Our study contributes to addressing these challenges by presenting the complete chloroplast genome of *Ranunculus* cf. *penicillatus*, a taxon widely recognized for its phenotypic plasticity and complex hybrid origin [1,20]. The availability of a complete plastid genome provides a stable molecular framework for investigating evolutionary relationships in a taxonomically difficult group and enables a more refined understanding of plastid genome structure and conservation within the genus.

The chloroplast genome of *R.* cf. *penicillatus* exhibits a typical angiosperm quadripartite structure, with conserved gene content and organization, including 112 genes, 78 of which encode proteins. These values are consistent with previous findings in *Ranunculus* and broader Ranunculaceae lineages [6,20,21,22]. Despite overall conservation, our comparative analyses revealed subtle structural differences, particularly in the expansion of inverted repeat (IR) regions in aquatic taxa such as *R. trichophyllus*, *R. mongolicus*, and *R.* cf. *penicillatus*. Such IR expansions, although not universal across the genus, appear more frequently among aquatic species, suggesting a possible association with environmental adaptation or genomic plasticity in response to aquatic habitats [23].

IR expansion may hold phylogenetic relevance, as it reflects lineage-specific genomic events that potentially influence gene stability and expression patterns. In plastid genomes, the IR regions are known to contain genes involved in core cellular functions, including rRNA and tRNA genes. The expansion of these regions might, therefore, contribute to enhanced functional robustness or adaptive potential under fluctuating aquatic conditions [23,24,25]. While the evolutionary drivers of IR length variation in *Ranunculus* remain speculative, this feature could be explored further in relation to environmental gradients, reproductive strategies, or life-history traits.

Phylogenetic reconstruction based on complete chloroplast genome sequences positions *Ranunculus* cf. *penicillatus* in a strongly supported clade with *R. trichophyllus*, *R. kadzusensis*, *R. mongolicus*, and *R. bungei* taxa that share morphological and ecological similarities. This result not only corroborates earlier hypotheses based on morphology and molecular markers but also reinforces the idea that aquatic *Ranunculus* species represent a distinct evolutionary lineage within the genus [6,21,26]. In contrast, terrestrial species cluster separately, underscoring the ecological and genetic divergence between aquatic and terrestrial clades.

These findings also have important implications for understanding the evolutionary origins of *Ranunculus* cf. *penicillatus*. As suggested by Cook (1966) [1] and later supported by Lansdown (2007) [27], this taxon likely represents a segmental allopolyploid derived from multiple hybridization events involving species such as *R. fluitans*, *R. trichophyllus*, and *R. aquatilis*. The chloroplast genome, which is typically maternally inherited in angiosperms, provides a useful window into maternal lineage history. The phylogenetic placement of *Ranunculus* cf. *penicillatus* alongside *R. trichophyllus*, *R. kadzusensis*, and *R. mongolicus* suggests that these species may share a common maternal ancestor, or that recurrent hybridization has resulted in cytoplasmic homogenization within this clade.

Furthermore, the conservation of genome size, structure, and gene content across aquatic taxa may reflect recent divergence or a high degree of genetic cohesion, which is consistent with the idea that the *Batrachium* section is an evolutionarily young group [1]. However, despite such genomic similarities, the presence of complex morphological variation and frequent hybridization indicates that speciation processes in aquatic *Ranunculus* are ongoing and likely influenced by both ecological factors and reproductive dynamics. Future studies integrating chloroplast data with nuclear and epigenomic markers could further elucidate the reticulate evolution and speciation patterns in this group.

From a conservation perspective, our findings underscore the value of plastid genomes for taxonomic clarification and biodiversity assessment. Aquatic plants are particularly vulnerable to habitat fragmentation, eutrophication, and hydrological alterations [28,29,30,31]. Misidentification or taxonomic ambiguity can hinder conservation efforts by obscuring the true distribution and genetic distinctiveness of taxa. This study provides a genomic reference sequence for *Ranunculus* cf. *penicillatus* and highlights its evolutionary position, creating a foundation for future conservation strategies. These strategies may include genetic monitoring as well as in situ or ex situ management of declining populations. The availability of organelle genomic resources for species provides the opportunity to develop new markers that can be used to evaluate biogeographical hypotheses and assess how species respond to changing environments [32].

In conclusion, the complete chloroplast genome of *Ranunculus* cf. *penicillatus* enriches the genomic resources available for the genus *Ranunculus* and offers new insights into the phylogenetics, taxonomy, and evolutionary biology of aquatic species within the section *Batrachium*. Our findings highlight the importance of integrating structural genome features with phylogenomic analysis to resolve complex taxonomic groups and to support effective biodiversity conservation in freshwater ecosystems.

## 4. Materials and Methods

Fresh leaf material was collected from a natural population of *Ranunculus* cf. *penicillatus*. in the Skroblus River (Lithuania) (54°00′48.0” 24°17′23.7”) in July 2021, identified morphologically according Cook, 1966 [1] and verified by sequencing (NCBI GenBank No. PV690257). The voucher specimens were deposited at the Vilnius University herbarium (WI) (No. P33603). Approximately 100 mg of fresh leaf tissue was ground manually in a mortar with liquid nitrogen, and DNA was extracted using the CTAB method as described previously [33]. The DNA samples were initially checked with 1.0% agarose gel electrophoresis before being sent to Thermo Fisher Scientific Baltics in Vilnius, Lithuania, for library preparation and sequencing through next-generation methods.

Whole-genome sequencing was performed using the Illumina NovaSeq (Ilumina, Inc., San Diego, CA, USA) platform. Approximately 9.0 GB of raw data were generated from the paired-end reads of 150 bp. The raw reads were filtered to remove adapters and low-quality sequences with Qualimap v. 2.3 [34]. Clean reads were assembled de novo using the same software with default parameters, and assembly quality was assessed through read mapping and coverage analysis using Qualimap. The coverage of *Ranunculus* cf. *penicillatus* was 2271×. The complete chloroplast genome was annotated using GeSeq v. 2.03 [35], with manual curation in Geneious Prime v. 2025.0 to verify gene boundaries and pseudogenes. Circular genome maps were generated using CPGAVAS2 (accessed on 2 July 2024) [36]. To assess structural variation and evolutionary relationships, we compared the assembled genome to other publicly available chloroplast genomes from the genus *Ranunculus*. We selected 4 other taxa that are aquatic and 4 terrestrial *Ranunculus* species to compare the chloroplaste genome and to make the same analysis. Phylogenetic analyses were performed using maximum likelihood (ML) based on whole-chloroplast genome alignments. Maximum likelihood trees were built with 1000 bootstrap replicates.

## Figures and Tables

**Table 1:** Chloroplast genome features of the nine samples of *Ranunculus* [2,6].

Species/Features	Genome Size (bp)	LSC Length (bp)	SSC Length (bp)	IR Length (bp)	Total GC Content (%)	Total Number of Genes	Protein Encoding
*Ranunculus* cf. *penicillatus*	158,313	84,937	17,635	27,849	36.80%	112	78
*R. trichophyllus*	158,314	84,945	17,635	27,853	36.80%	112	78
*R. mongolicus*	158,309	84,974	17,637	27,849	36.80%	112	78
*R. bungei*	156,082	85,430	19,948	25,352	36.70%	112	78
*R. kadzusensis*	158,301	84,973	17,638	27,845	37.83%	112	78
*R. monophyllus*	155,973	85,345	19,790	25,419	36.80%	112	78
*R. polyrhizos*	156,000	85,414	19,677	25,455	36.80%	112	78
*R. tanguticus*	156,186	85,627	19,785	25,387	36.80%	112	78
*R. sceleratus*	156,329	85,840	19,885	25,302	41.22%	112	78

## Data Availability

All other relevant data are available from the corresponding author upon reasonable request.
